# The Transcriptional Coactivator *DEAD/H Box 5 (DDX5)* Gene Is a Target of the Transcription Factor E2F1 Deregulated from the Tumor Suppressor pRB

**DOI:** 10.3390/genes16080929

**Published:** 2025-08-01

**Authors:** Rinka Nakajima, Yaxuan Zhou, Mashiro Shirasawa, Mariana Fikriyanti, Ritsuko Iwanaga, Andrew P. Bradford, Kenta Kurayoshi, Keigo Araki, Kiyoshi Ohtani

**Affiliations:** 1 Department of Biomedical Sciences, School of Biological and Environmental Sciences, Kwansei Gakuin University, 1 Gakuen Uegahara, Sanda 669-1330, Japan; hnj51097@kwansei.ac.jp (R.N.); gtk53096@kwansei.ac.jp (Y.Z.); icf08267@kwansei.ac.jp (M.S.); hsj19688@kwansei.ac.jp (M.F.); 2 Department of Obstetrics and Gynecology, University of Colorado School of Medicine—Anschutz Medical Campus, 12700 East 19th Avenue, Aurora, CO 80045, USA; ritsuko.iwanaga@cuanschutz.edu (R.I.); andy.bradford@cuanschutz.edu (A.P.B.); 3 Division of Molecular Genetics, Cancer Research Institute, Kanazawa University, Kakuma-Machi, Kanazawa 920-1192, Japan; kuraken0901@gmail.com; 4 Department of Morphological Biology, Ohu University School of Dentistry, 31-1 Misumido Tomitamachi, Koriyama 963-8611, Japan; k-araki@den.ohu-u.ac.jp

**Keywords:** DDX5, E2F1, pRB, gene expression, promoter analysis

## Abstract

Background: DEAD/H box 5 (DDX5) serves as a transcriptional coactivator for several transcription factors including E2F1, the primary target of the tumor suppressor pRB. E2F1 physiologically activated by growth stimulation activates growth-related genes and promotes cell proliferation. In contrast, upon loss of pRB function due to oncogenic changes, E2F1 is activated out of restraint by pRB (deregulated E2F1) and stimulates tumor suppressor genes such as *ARF*, which activates the tumor suppressor p53, to suppress tumorigenesis. We have recently reported that DDX5 augments deregulated E2F1 activity to induce tumor suppressor gene expression and apoptosis. During the analyses, we noted that over-expression of E2F1 increased DDX5 expression, suggesting a feed forward loop in E2F1 activation through DDX5. Objective: We thus examined whether the *DDX5* gene is a target of deregulated E2F1. Method: For this purpose, we performed promoter analysis and ChIP assay. Result: The DDX5 promoter did not possess typical E2F binding consensus but contained several GC repeats observed in deregulated E2F1 targets. Insertion of point mutations in these GC repeats decreased responsiveness to deregulated E2F1 induced by over-expression of E2F1, but scarcely affected responsiveness to growth stimulation. ChIP assays showed that deregulated E2F1 induced by over-expression of E2F1 or expression of E1a, which binds pRB and releases E2F1, bound to the *DDX5* gene, while physiological E2F1 induced by growth stimulation did not. Conclusions: These results suggest that the *DDX5* gene is a target of deregulated E2F1, generating a feed forward loop mediating tumor suppressive E2F1 activity.

## 1. Introduction

DEAD/H box 5 (DDX5) was identified as a cellular protein, which cross-reacted with antibody against SV40T, and functions as an RNA helicase [1,2]. DDX5 contributes to RNA metabolism and processing, such as pre-mRNA splicing, rRNA and miRNA processing, thereby playing crucial roles in a plethora of biological processes [3,4,5,6,7]. DDX5 also functions as a coactivator for several transcription factors, such as estrogen receptor α (ERα) [8], androgen receptor [9], Runt-related transcription factor 2 (Runx2) [10], β-catenin [11], c-Myc [12], nuclear factor-kappa B (NF-κB) [13,14] and E2F1 [15], thereby contributing to cell proliferation. Enhanced expression of DDX5 is often observed in several types of cancers, such as colon, prostate, and breast cancers and is believed to contributes to proliferation of cancer cells by facilitating growth-related gene expression [8,16,17,18].

The transcription factor E2F1 is a crucial target of the tumor suppressor pRB that regulates important biological events such as cell proliferation, by activating growth-related genes, and apoptosis, by activating pro-apoptotic genes [19,20,21,22]. Physiological E2F1 activity, induced by growth stimulation through inactivation of pRB by cyclin/CDK-mediated phosphorylation, activates a series of growth-related genes, leading to cell proliferation [19,23,24,25]. In contrast, E2F1 when activated out of restraints by pRB upon loss of pRB functions due to oncogenic changes (deregulated E2F1), activates pro-apoptotic genes in addition to growth-related genes, such as *ARF*, the upstream regulator of p53, thereby carrying out crucial roles in tumor suppression [26]. Hence, in almost all cancers, in addition to the RB pathway the p53 pathway is also disabled by oncogenic changes including deletion or mutation of ARF or p53, or Hdm2 over-expression, which ubiquitinates p53 for proteasome-mediated degradation [20], enabling cancer cells to survive. Of note, deregulated E2F1 can induce apoptosis not depending on p53 by activating other tumor suppressor genes such as *TAp73* and *Bim* [27,28,29], which could serve as a means to target p53-deficient cancer cells for treatment.

It is generally accepted that DDX5 contributes to cancer cell proliferation by facilitating RNA metabolism and enhancing activity of growth-promoting transcription factors by functioning as a transcriptional coactivator [30,31,32]. However, DDX5 is also reported to enhance the activity of the tumor suppressor p53 in the DNA damage response, suggesting a role of DDX5 for tumor suppression [33,34]. Accordingly, our recent analysis of the effects of DDX5 on E2F1 to regulate tumor suppressor genes demonstrated that DDX5 augments E2F1 activity to induce tumor suppressor gene expression and apoptosis [35]. Moreover, E2F1 could induce apoptosis in a p53-null cancer cell line through a p53-independent pathway, which was also enhanced by DDX5. These observations underscore the potential of p53-independent pathways in treatment of p53-disabled cancers. During the course of these analyses, we found that over-expression of E2F1 or expression of adenovirus E1a, which binds to pRB and releases E2F1, enhanced DDX5 expression, suggesting that the *DDX5* gene itself is a target of deregulated E2F1. In this context, the *DDX5* gene targeted by deregulated E2F1, would form a positive feed forward loop mechanism contributing to E2F1-mediated tumor suppression. Thus, herein we performed DDX5 promoter analysis. Our results show that the *DDX5* gene is indeed a target of deregulated E2F1, but not physiological E2F1 induced by growth stimulation.

## 2. Materials and Methods

### 2.1. Cell Culture

Human foreskin fibroblasts (HFFs, obtained from ATCC), human embryonic kidney HEK293A cells and rat embryonic fibroblast REF52 cells were cultured in Dulbecco’s modified Eagle medium (DMEM) supplemented with 10% fetal calf serum (FCS). To synchronize the cell cycle in the resting state, HFFs and REF52 cells were cultured under the serum-starved condition (DMEM containing 0.1% FCS) for 2 days and 3 days, respectively. For serum simulation, the cells were then re-stimulated with serum by adding FCS to a final concentration of 20% or replacing the medium with DMEM containing 10% FCS, respectively.

### 2.2. Plasmids

pDDX5-Luc(−1259) was constructed as follows: −1259~+730 (transcription start site as +1) region of DDX5 promoter was amplified by nested PCR using HFF genomic DNA as the template using KOD FX Neo (TOYOBO) and the following primer sets:

1st PCR

Fw: 5′-CTGTTGGGAAAAAGGGGGAAAGAT-3′

Rv: 5′-GCCGGGCGGGGTAACAA-3′

2nd PCR

Fw: 5′-AGTggtaccTCTCTGGTGCCGGAAAAAGT-3′

Rv: 5′-AGTctcgagTTGGCGGGGAACGAAGTAT-3′

The amplified PCR product was treated with *Kpn*I and *Xho*I, and inserted into *Kpn*I and *Xho*I sites of pGL3-Basic (Promega). Point mutants of pDDX5-Luc(−1259) were constructed by PCR-based mutagenesis. pGL3-Promoter (Promega), pCDC6-Luc(−570), pCMV-b-gal and pENTR-E2F1 have been described previously [36].

### 2.3. Transfection and Reporter Assay

HFFs and RFF52 cells were transfected using PEI Max (Polysciences) with the proportion of DNA versus PEI 1:3. Luciferase assay was performed as described previously [36]. For co-transfection experiments, HFFs were expanded into 60 mm dishes (split ratio 1:10). After 16 h, reporter and effector plasmids were transfected using PEI Max along with the internal control pCMV-β-gal to monitor transfection efficiency. After 28 h, the cells were harvested. For serum stimulation experiments, REF52 cells were split (1:15) into 60 mm dishes. After 20 h, the medium was changed to DMEM containing 0.1% FCS for 2 days to arrest the cells in the quiescent stage. The synchronous REF52 cells were transfected with reporter and pCMV-β-gal plasmids using PEI Max. After 20 h, the cells were washed with PBS and further cultured in DMEM containing 0.1% or 10% FCS, respectively, and were harvested after 20 h. Luciferase activities were measured using the Luciferase Assay System (Promega) and adjusted to β-galactosidase activities as an internal control. All assays were repeated at least three times and the results are shown as means ± SD.

### 2.4. Infection with Recombinant Adenovirus

Ad-E2F1, Ad-FLAG-E2F1, Ad-12SE1a(Δ2-11), and Ad-Con were described previously [29,36]. Large scale preparation of the viruses were performed in HEK293A cells and purified by 2 rounds of ultracentrifuge using a discontinuous CsCl density gradient. The titer of purified virus was measured by immunofluorescence staining using rabbit polyclonal antibodies against adenovirus *E2* gene product. HFFs were infected with the viruses for 1 h in 1 mL or 0.5 mL DMEM for a 100 mm dish or 60 mm dish, respectively, containing the indicated multiples of infection (MOI) of the virus. The cells were further cultured in DMEM containing 0.1% or 10% FCS for the indicated times before harvesting.

### 2.5. Quantitative Reverse Transcription (qRT)-PCR

qRT-PCR was performed as described previously [35]. Total RNA extraction was performed using Isogen II (Nippon Gene, Chiyoda, Tokyo, Japan). First strand cDNA synthesis was performed using PrimeScript 1st strand cDNA Synthesis Kit (Takara Bio, Kusatsu, Shiga, Japan). Quantitative PCR was performed using PowerUp SYBR Green Master Mix (applied biosystems, Carlsbad, CA, USA) and QuantStudio 3 (applied biosystems, Carlsbad, CA, USA). Gene-specific primer sets for the *E2F1*, *DDX5* and *GAPDH* genes have been described previously [35]. The results were adjusted by that of *GAPDH* as an internal control.

### 2.6. Immunoblot Analysis

Immunoblot analysis was performed as described previously [37]. The antibodies used were anti-DDX5 (ab126730, abcam, Cambridge, UK, 1:500), anti-E2F1 (sc-251, Santa Cruz Biotechnology, Dallas, TX, USA, 1:250), anti-E1a (clone M58, 554155, BD Pharmingen, Franklin Lakes, NJ, USA, 1:500), anti-β-actin (A1978, SIGMA, Tokyo, Japan, 1:2000), anti-mouse IgG-HRP (Jackson, Scottsdale, CA, USA, 1:1000) and anti-rabbit IgG-HRP (Cell signaling, Danvers, MA, USA, 1:2500). The membranes were treated with ImmunoStar LD (Fujifilm, Tokyo, Japan) and the signals were detected using LAS4000 (GE Healthcare, Chicago, IL, USA). Quantification of the signals was performed by ImageJ version 1.51 (NIH, Bethesda, MD, USA) and adjusted by that of β-actin as an internal control.

### 2.7. Chromatin Immunoprecipitation (ChIP) Assay

ChIP assay was performed as described [29]. Antibodies used were anti-E2F1 (sc-251X, Santa Cruz) and anti-HA (sc-7392, Santa Cruz) as a negative control. Gene-specific primer sets for the *CDC6* and *β-actin* genes have been described previously [29]. Primer sets for the *DDX5* gene are listed below. Two mL of immunoprecipitated samples were amplified 25 cycles for first round PCR. Two mL of first round PCR products were further amplified for 18 cycles for the second round. Input was one 10th of the lysates.

*DDX5* primers

Fw: 5′-GTGGCGCGGGGAGGGGTGAAA-3′

Rv: 5′-GGGGGCGGCAGCGGAGGAAG-3′

### 2.8. Statistical Analysis

All experiments were repeated at least three times. Data are shown as means ± SD. Statistical analysis was carried out using Student’s *t*-test and Bonferroni correction. *p* or *Q* value < 0.05 was considered significant.

## 3. Results

### 3.1. Deregulated E2F1 and Growth Stimulation Induced DDX5 Gene Expression in Human Normal Fibroblasts

**Figure 1:**
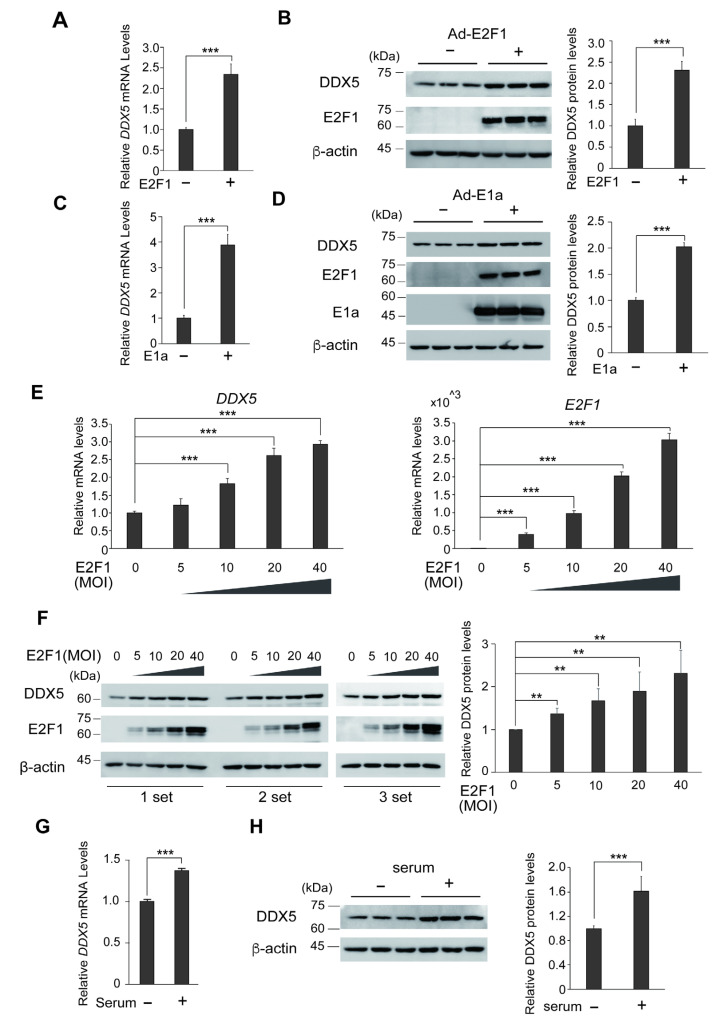
(**A**) Deregulated E2F1 enhanced *DDX5* gene expression. HFFs were infected with Ad-E2F1 or Ad-Con as a control (MOI: 100), further cultured for 24 h and harvested. The *DDX5* mRNA expression levels were examined by qRT-PCR and adjusted by that of *GAPDH* as an internal control. ***: *p* < 0.01. (**B**) Deregulated E2F1 enhanced DDX5 protein expression. The DDX5 and E2F1 protein levels were examined by Western blot analysis under the same condition as in (**A**). The graph shows the quantification of DDX5 expression adjusted by that of β-actin as an internal control. ***: *p* < 0.01. (**C**) Endogenous deregulated E2F induced *DDX5* gene expression. HFFs were infected with Ad-12SE1a(Δ2-11) or Ad-Con as a control (MOI: 100), further cultured for 48 h in DMEM containing 0.1% FCS and harvested. The levels of *DDX5* mRNA were examined by qRT-PCR as above. ***: *p* < 0.01. (**D**) Endogenous deregulated E2F induced DDX5 protein expression. DDX5, E2F1 and E1a protein levels were examined by Western blot analysis under the same condition as in (**C**). The graph shows the quantification of DDX5 expression adjusted by that of β-actin as an internal control. (**E**) DDX5 expression was induced by over-expression of E2F1 in a dose dependent manner. HFFs were infected with Ad-FLAG-E2F1 (MOI: 5, 10, 20, 40), further cultured for 24 h and harvested. The levels of *DDX5* and *E2F1* mRNA were examined by qRT-PCR as above. ***: *Q* < 0.01. (**F**) The DDX5 and E2F1 protein levels were examined by Western blot analysis under the same condition as in (**E**). The graph shows the quantification of DDX5 expression normalized by that of β-actin as an internal control. **: *Q* < 0.05, ***: *Q* < 0.01. (**G**) Growth stimulation increases endogenous *DDX5* gene expression in HFFs. HFFs were starved of serum for 72 h, restimulated with serum for 20 h and harvested. The levels of *DDX5* mRNA were examined by qRT-PCR and adjusted by that of *GAPDH* as an internal control. ***: *p* < 0.01. (**H**) DDX5 protein levels were examined by Western blot analysis under the same condition as in (**G**). The graph shows the quantification of DDX5 expression normalized by that of β-actin as an internal control. ***: *p* < 0.01.

We first confirmed that the expression of *DDX5* gene, at the mRNA and protein levels, is induced by deregulated E2F1, which activates pro-apoptotic E2F target genes, in human normal fibroblasts (HFFs). To generate deregulated E2F1, we over-expressed E2F1 or expressed adenovirus E1a, which binds to pRB and releases E2F1, using recombinant adenoviruses. HFFs were infected with adenovirus expressing E2F1 (Ad-E2F1) or E1a (Ad-12SE1a(Δ2-11)) (MOI 100) and harvested after 24 h. Over-expression of E2F1 enhanced *DDX5* mRNA level 2.3-fold (Figure 1A), and DDX5 protein level 2.3-fold (Figure 1B). Moreover, endogenous deregulated E2F1, which was generated by E1a expression, enhanced *DDX5* mRNA level 3.9-fold (Figure 1C) and DDX5 protein level 2.0-fold (Figure 1D). We also examined whether *DDX5* gene expression depends on the amount of E2F1 expression. HFFs were similarly infected with Ad-FLAG-E2F1 (MOI 5, 10, 20, 40) and harvested after 24 h. *DDX5* mRNA levels were significantly increased by E2F1 in a dose dependent manner (Figure 1E left panel). The levels of E2F1 expression corresponding to viral MOI were confirmed (Figure 1E right panel). DDX5 protein levels were also increased by E2F1 in a dose dependent manner (Figure 1F). These results suggest that the *DDX5* gene is a target of deregulated E2F1. To determine if the *DDX5* gene is also a target of physiological E2F, we also examined whether expression of *DDX5* is induced in response to serum stimulation of HFFs. HFFs were starved of serum for 72 h and restimulated with serum at final concentration of 20% for 20 h and harvested. Serum stimulation induced *DDX5* mRNA level 1.4-fold (Figure 1G) and the protein level 1.6-fold (Figure 1H), suggesting the possibility that growth stimulation activates the *DDX5* gene through physiological E2F1 or other growth-related transcription factor(s).

### 3.2. Over-Expression of E2F1 Activates DDX5 Promoter

**Figure 2:**
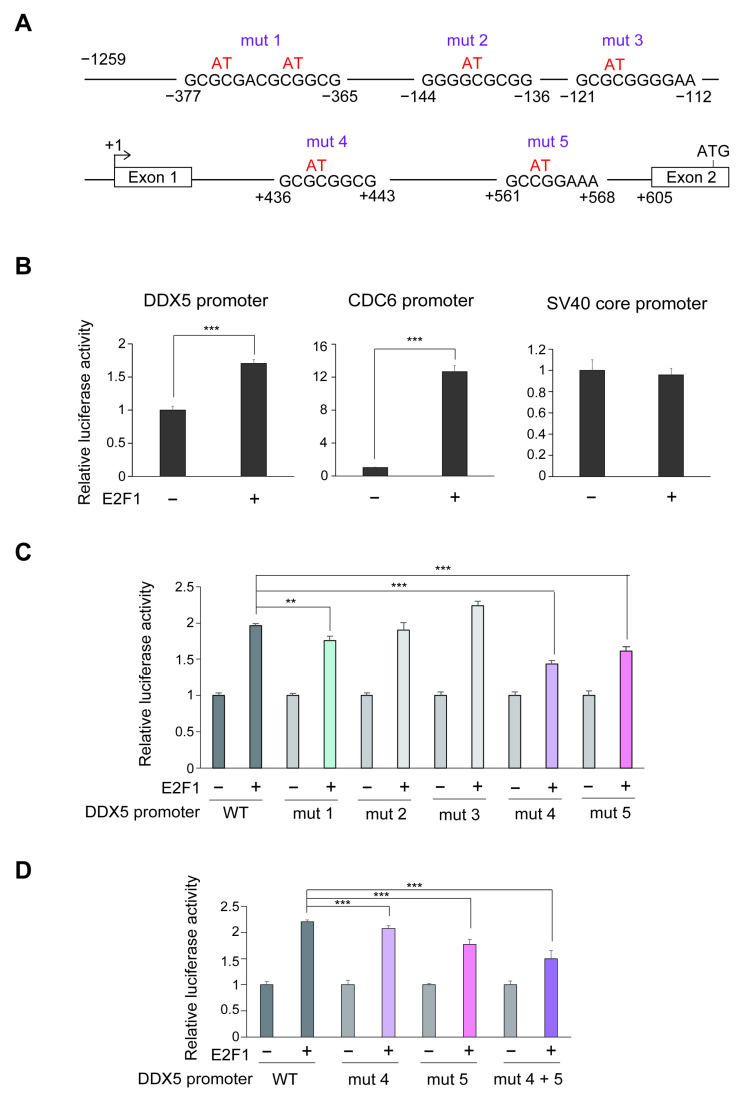
(**A**) Schematic presentation of cloned DDX5 promoter (−1259~+730). Sequences shown are 5 candidates for E2F1-responsive sequences, which are rich in GC repetitive sequences, and mutation of these sequences. (**B**) E2F1 over-expression activated the DDX5 promoter. HFFs were transfected with DDX5 reporter plasmid (1.7 μg) and β-galactosidase expression plasmid (0.3 μg) as an internal control with or without E2F1 expression vector (10 ng), further cultured for 28 h and harvested. CDC6 and SV40 core reporter plasmids were used as positive and negative controls, respectively. Luciferase activities were normalized by β-galactosidase activities as an internal control. Relative activities are shown as a graph. ***: *p* < 0.01. (**C**) Mutation of GC repetitive sequences reduced E2F1-responsiveness. Responsiveness of each mutant DDX5 promoter to E2F1 over-expression was similarly examined as in (**B**). For each reporter, the luciferase activities are shown relative to that in the absence of E2F1 expression as 1. **: *Q* < 0.05, ***: *Q* < 0.01. Reporters with significantly reduced responsiveness are shown in color bars. (**D**) Combination of point mutations further reduced E2F1-responsiveness. Responsiveness of mutant DDX5 promoter with combination of mut 4 and mut 5 to E2F1 was similarly examined as above. ***: *Q* < 0.01.

To further analyze whether the *DDX5* gene is a target of deregulated and physiological E2F1, we performed DDX5 promoter analysis. We first examined the sequence of the DDX5 promoter region to identify E2F1 responsive elements; either canonical E2F binding consensus sites observed in growth-related targets or atypical GC repeats, which specifically respond to deregulated E2F1 but not to physiological E2F1 induced by growth stimulation [29,36]. We did not find typical E2F binding consensus sequences but identified 5 candidate GC repeat deregulated E2F1 responsive elements at −377/−365, −144/−136, −121/−112, +436/+443 and +561/+568 of DDX5 promoter, with the transcription start site designated as +1. The *DDX5* gene possesses a translation initiation codon in the 2nd exon at +748. Thus, to examine the responsiveness to deregulated E2F1, we cloned the DDX5 promoter −1259 to +730 containing these candidates upstream of the translational initiation site, into a luciferase vector (Figure 2A). Over-expression of E2F1 activated the DDX5 promoter and, as a positive control, the CDC6 promoter, a known growth-related E2F1 target gene, but not the SV40 core promoter, a negative control (Figure 2B). This result suggests that −1259 to +730 region of the DDX5 promoter contains atypical E2F1 responsive elements. To identify specific E2F1 response elements, we next introduced point mutations into each of the GC repeats, designated mut 1 (−377/−365), mut 2 (−144/−136), mut 3 (−121/−112), mut 4 (+436/+443) and mut 5 (+561/+568) (Figure 2A). Among these mutant reporters, mut 1, mut 4 and mut 5 showed decreased responsiveness to over-expression of E2F1 compared to the wild type promoter (WT) (Figure 2C). Combination of mut 4 and mut 5, further decreased the E2F1 response, but some E2F dependent activity remained (Figure 2D). These results suggest that the DDX5 promoter possesses several E2F1 responsive elements, which co-operatively contribute to E2F1-induced gene transcription.

### 3.3. Physiological E2F1 Induced by Growth-Stimulation Does Not Activate DDX5 Promoter

**Figure 3:**
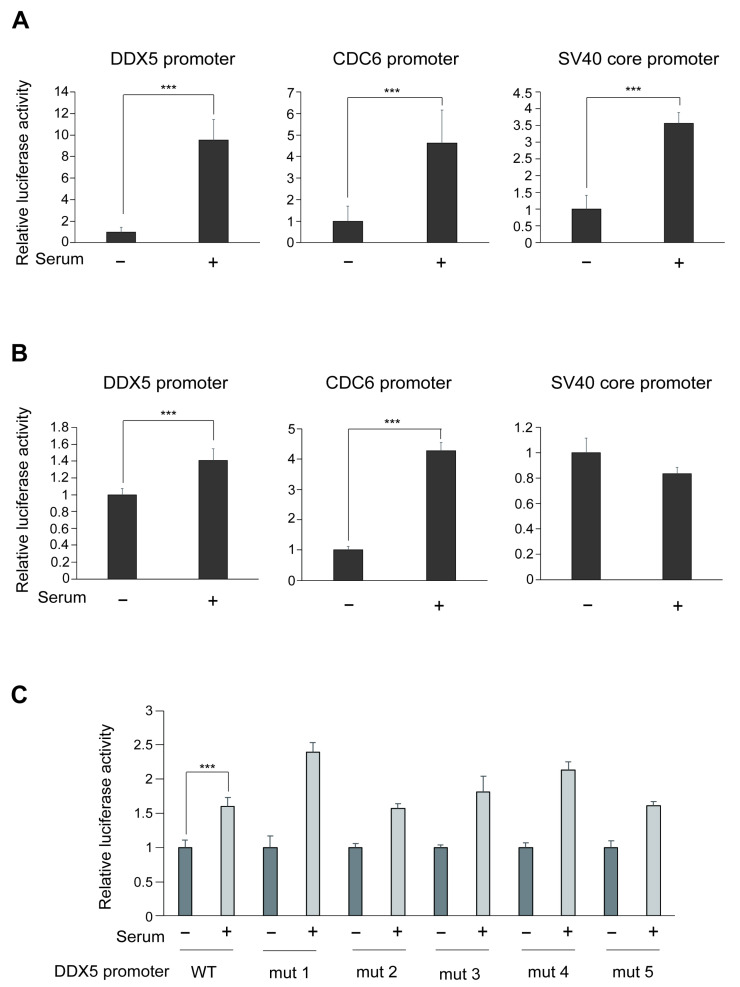
(**A**) Responsiveness of the DDX5 promoter (−1259~+730) to serum stimulation in HFFs. The cells were transfected with DDX5 reporter plasmid (1.7 μg) and β-galactosidase expression plasmid (0.3 μg) as an internal control. After 20 h, the cells were washed with PBS and further cultured in DMEM containing 0.1% FCS for 2 days. Serum was added at the final concentration of 20% and harvested after 20 h. CDC6 and SV40 core promoters were used as positive and negative controls, respectively. Luciferase activities were assayed and normalized by that of β-galactosidase as an internal control, and relative activities are shown as a graph. ***: *p* < 0.01. (**B**) Responsiveness of the DDX5 promoter (−1259~+730) to serum stimulation in REF52 cells. The cells were starved of serum for 2 days, transfected with the reporters as same as in (**A**). The cells were washed with PBS and further cultured under the starved condition or in the presence of serum (10% FCS) and harvested after 20 h. Luciferase activities were similarly examined as in (**A**). ***: *p* < 0.01. (**C**) Mutation of the GC repetitive sequences did not compromise serum-responsiveness. Responsiveness of each mutant DDX5 promoter to serum stimulation was similarly examined as in (**A**). ***: *p* < 0.01.

Since *DDX5* gene expression was induced by serum stimulation of HFFs (Figure 1G,H), we examined whether DDX5 promoter is activated by physiological E2F1 induced by serum stimulation in HFFs. HFFs were transfected with the DDX5 reporter (−1259/+730) or CDC6 reporter plasmid, as a positive control, or SV40 core reporter as a negative control. After 20 h, the cells were washed with PBS and further cultured in DMEM containing 0.1% FCS for 2 days. Serum was added at the final concentration of 20% and harvested after 20 h. Serum stimulation activated the DDX5 promoter and CDC6 promoter but also the negative control SV40 core promoter (Figure 3A). Since serum stimulation activated all promoters in HFFs, it was difficult to examine responsiveness to physiological E2F induced by serum stimulation under this condition. We thus examined the responsiveness to serum stimulation in rat embryonic fibroblast REF52 cells, which are more efficiently serum-starved and re-stimulated than HFFs, and have been successfully utilized in previous experiments [38]. REF52 cells were starved of serum for 2 days, transfected with the DDX5, CDC6 or SV40 core reporter. After 20 h, the cells were washed with PBS and further cultured in DMEM containing 0.1% or 10% FCS. Serum stimulation activated the DDX5 promoter and CDC6 promoter but not the SV40 core promoter, suggesting that DDX5 promoter responds to serum stimulation (Figure 3B). We next examined whether this enhancement is mediated through physiological E2F1 using the point mutants of DDX5 promoter as in Figure 2A. Serum stimulation activated each mutant promoter to the similar extents as DDX5 (WT) promoter (Figure 3C), i.e., mutation of the E2F1 GC repeat elements had no significant effect on serum induced DDX5 promoter activity. These results strongly suggest that the DDX5 promoter is selectively activated by deregulated E2F1, but not physiological E2F1 induced by serum stimulation, through the GC repeat sequences.

### 3.4. The DDX5 Gene Is a Target of Deregulated E2F1

**Figure 4:**
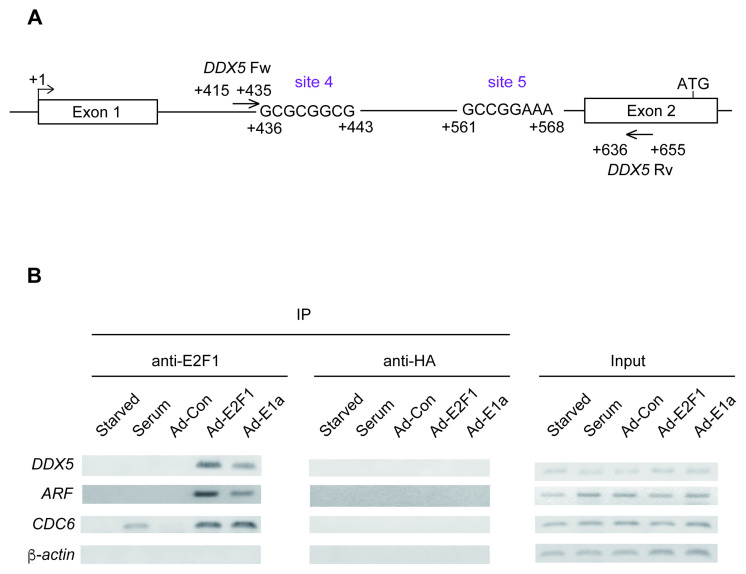
(**A**) Schematic presentation of primer localization for the *DDX5* gene. The primers were designed to flank the mut 4 and mut 5 sites in DDX5 promoter. (**B**) The *DDX5* gene specifically bound deregulated E2F1. HFFs were starved of serum, restimulated with serum or infected with adenovirus expressing E2F1 or E1a, and further cultured for 24 h. ChIP assay was performed using anti-E2F1 antibody and anti-HA antibody as a negative control. The *ARF* and *CDC6* genes were used as positive controls for binding of deregulated and physiological E2F1, respectively, and the *b-actin* gene was used as a negative control.

We finally examined whether the *DDX5* gene was bound by deregulated E2F1 but not by physiological E2F1 using chromatin immunoprecipitation (ChIP) assay. We designed DDX5 target gene primers flanking the sites of both mut 4 (+436/+443) and mut 5 (+561/+568) (Figure 4A), mutation of which significantly reduced responsiveness to over-expressed E2F1 (Figure 2D). We used the *ARF* gene as a positive control for deregulated E2F1 targets, the *CDC6* gene as a positive control for physiological E2F1 targets induced by serum stimulation, and the *β-actin* gene as a negative control. The results showed that deregulated E2F1, induced by over-expression of E2F1 or expression of E1a, bound to the *DDX5* gene (Figure 4B). In contrast, binding of physiological E2F1, induced by serum stimulation, to the putative E2F1 response elements in the DDX5 promoter was not detected. These results suggest that the *DDX5* gene is a target of deregulated E2F1 but not physiological E2F1.

## 4. Discussion

**Figure 5:**
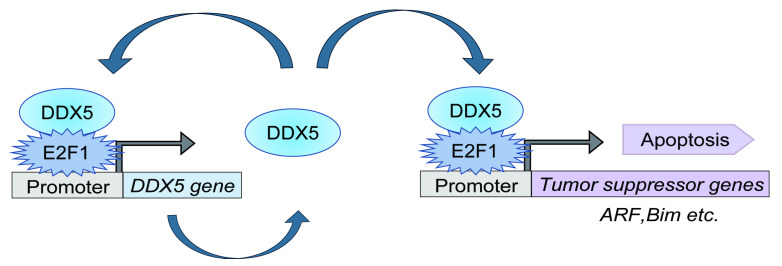
A model for the feed forward loop in deregulated E2F1 activity through induction of DDX5. DDX5 enhances deregulated E2F1 activity, which in turn activates the *DDX5* gene, thereby forming a feed forward loop in deregulated E2F1 activity that activates tumor suppressor genes.

In this study, we found that the *DDX5* gene is a target of deregulated E2F1 but not physiological E2F1 induced by growth stimulation. DDX5 expression was induced not only by deregulated E2F1, generated by over-expression of E2F1 or adenovirus E1a, which binds pRB and releases E2F1, but also by serum stimulation in human normal fibroblast HFFs (Figure 1). The DDX5 promoter was activated by over-expression of E2F1 and serum stimulation, suggesting that induction of *DDX5* gene expression is mediated at the transcriptional level. The DDX5 promoter did not exhibit typical E2F binding consensus sequences (TTTCGCGC), observed in growth-related target genes usually located within +100 bp from the transcription start site, but possessed GC repetitive sequences, characteristic of deregulated E2F1 target genes, such as *ARF* and *BIM* [29]. Introduction of point mutations in these atypical elements reduced responsiveness to over-expressed E2F1 and in combination point mutations further reduced the promoter response, suggesting that these elements cooperatively contribute to responsiveness to over-expression of E2F1. However, these mutations did not compromise activation of the DDX5 promoter by serum stimulation, indicating that physiological E2F1 induced by serum stimulation does not activate the DDX5 promoter through these elements (Figure 2 and Figure 3). Moreover, ChIP assay showed that deregulated E2F1, generated by over-expression of E2F1 and expression of E1a, bound to the *DDX5* gene but, in contrast to the growth-related *CDC6* gene, physiological E2F1 did not bind to the putative E2F1-response elements of the *DDX5* gene (Figure 4). These results suggest that the *DDX5* gene is a target of deregulated E2F1 but not physiological E2F1 induced by growth stimulation. Together with our recent finding that DDX5 augments deregulated E2F1 activity [35], activation of the DDX5 promoter by deregulated E2F1 suggests the presence of a feed forward loop in tumor suppressive E2F1 activity (Figure 5). Indeed, down regulation of endogenous DDX5 expression by shRNA reduced deregulated E2F1-induction of tumor suppressor gene expression and apoptosis [35]. Activation of the *DDX5* gene by serum stimulation is likely mediated by transcription factors other than E2F. We searched putative transcription factor binding sequences, which could respond to growth stimulation, in the DDX5 promoter (−1259/+730) region by JASPAR 2024, and identified several cAMP responsive element binding protein 1 (CREB1) binding sites (+142/+149, +355/+362, +499/+501, +620/+627) with more than 80% match. Identification of responsive element(s) to growth stimulation in the DDX5 promoter would be a future area of research.

In this study, we did not examine whether serum stimulation induced expression of E2F1 or compared the amount of E2F1 induced by serum stimulation with those induced by over-expression of E2F1 or expression of adenovirus E1a. Thus one may speculate the reason why serum stimulation did not activate DDX5 promoter through the GC-rich sequence is that the amount of E2F1 induced by serum stimulation was less than that induced by over-expression of E2F1 or expression of adenovirus E1a. However, we do not think this is the case since we have identified that deregulated E2F1 has distinct activity from physiologically induced E2F1 activity, which cannot be explained by the amount of E2F1. We previously reported that deregulated E2F1 activates tumor suppressor genes such as the *ARF* and *Bim* [29,36], which are not activated by physiological E2F1 induced by serum stimulation. The differential effects between deregulated E2F1 and physiological E2F1 cannot be explained by the different amount of E2F1, which has been suggested by the threshold model [39], as shown by knockdown of E2F1-heterodimeric partner DP [40]. Knockdown of DP compromised physiological E2F1 activity but did not affect deregulated E2F1 activity at all. This indicates that deregulated E2F1 has distinct activity from physiological E2F1 that does not depend on DP. The *E2F1* gene expression is induced by serum stimulation through E2F itself [41] and serum stimulation induced E2F1 expression in the same cell line in our previous experiments [42]. Moreover, serum stimulation activated CDC6 promoter, one of growth-related E2F1 targets (Figure 3B), and induced binding of E2F1 to CDC6 promoter in ChIP assay (Figure 4B). Thus, it is well expected that serum stimulation induced E2F1 expression in this study. Taken together, the *DDX5* gene is thought to be a target of deregulated E2F1 but not physiological E2F1.

It is generally regarded that DDX5 promotes tumorigenesis by functioning as a transcriptional coactivator for growth-promoting transcription factors, thereby contributing to proliferation of cancer cells [30,31,32]. However, in our recent study, we clarified that DDX5 also contributes to deregulated E2F1-mediated tumor suppressor gene expression and apoptosis [35]. Physiological E2F1 induced by growth stimulation activates growth-related genes and promotes cell proliferation [19,23,24,25]. In contrast, deregulated E2F1 induced by loss of pRB function upon oncogenic changes activates not only growth-related genes but also tumor suppressor genes such as *ARF*, the upstream regulator of p53, thereby protecting cells from tumorigenesis. Accordingly, in almost all cancers, not only the RB pathway but also p53 pathway is disabled by oncogenic changes. In this regard, it is noteworthy that, in addition to *ARF*, deregulated E2F1 activates other tumor suppressor genes, such as *TAp73* and *Bim*, which can induce apoptosis independent of p53 [29]. We recently reported that E2F1 can induce apoptosis in a p53-null cancer cel line, in which DDX5 enhances deregulated E2F1 activity and E2F1-induced apoptosis independent of p53 [35]. Since the RB and p53 pathways are disabled in almost all cancers, E2F1’s ability to induce apoptosis independent of p53 is a fascinating means by which to approach targeting of p53-disabled cancer cells. Our finding that deregulated E2F1 activates the *DDX5* gene suggests a positive feed forward loop in deregulated E2F1 activity to suppress tumorigenesis, which may be an important component of such therapeutic interventions.

It is reported that, in several breast cancer cell lines, DDX5 is over-expressed and down regulation of DDX5 expression reduced expression of growth-related E2F target genes and suppressed proliferation of the cancer cells [15]. This observation suggests the possibility that there is a similar positive feed forward loop in E2F1 activity through DDX5 in the cancer cell lines. In almost all cancers, function of p53, to which DDX5 also functions as a coactivator, is disabled. Hence enhanced DDX5-E2F1 activity may be directed for cell proliferation in cancer cells. However, in our previous report [35], we showed that E2F1 can induce cell death in p53-null cancer cell line through p53-independent pathway and that DDX5 enhanced the E2F1-mediated cell death independent of p53. This suggests that enhanced DDX5-E2F1 activity may also support p53-independent E2F1-meciated cell death pathway, which is somehow not sufficient to induce cell death, in cancer cells. Whether DDX5-E2F1 feed forward loop also exists in cancer cells and, if it does, its contribution to cell proliferation or cell death would be important future issues to be addressed to approach p53-disabled cancer cells.

## Data Availability

The raw data supporting the conclusions of this article will be made available by the authors on request.

## Abbreviations

ARF, alternative reading frame; CDC6, cell division cycle 6; CDK, cyclin-dependent kinase; ChIP, chromatin immunoprecipitation; CMV, cytomegalovirus; CREB1, cAMP responsive element binding protein 1; DDX5, DEAD/H box 5; DMEM, Dulbecco’s modified Eagle medium; E2F, E2 transcription factor; ERa, Estrogen receptor a; FCS, fetal calf serum; GAPDH, glyceraldehyde-3-phosphate dehydrogenase; HA, hemagglutinin; Hdm2, human double minute 2; HEK293A, human embryonic kidney 293A; HFF, human foreskin fibroblast; HRP, horseradish peroxidase; MOI, multiples of infection; NF-kB, Nuclear factor-kappa B; PEI, polyethylenimine; pRB, retinoblastoma protein; RT, reverse transcription; Runx2, Runt-related transcription factor 2; SD, standard deviation; SV40, simian virus 40; TAp73, transactivating p73.
